# Impact of methicillin-resistant *Staphylococcus aureus* surveillance and decolonization in the NICU: the Texas children’s hospital experience

**DOI:** 10.1017/ash.2025.45

**Published:** 2025-02-24

**Authors:** Nahid Hiermandi, Catherine Foster, Judith Campbell, Krystal Purnell, Elizabeth Tocco, Tjin Koy, Kenneth Nobleza, Duc Nguyen, Lucila Marquez

**Affiliations:** 1 Division of Infectious Diseases, Department of Pediatrics, Baylor College of Medicine, Houston, TX, USA; 2 Department of Infection Prevention and Control, Texas Children’s Hospital, Houston, TX, USA; 3 Department of Pediatrics, Baylor College of Medicine, Houston, TX, USA

## Abstract

**Objective:**

To determine the impact of screening and decolonization on methicillin-resistant *Staphylococcus aureus* (MRSA) infection in a neonatal intensive care unit.

**Study Design:**

This is a single-center retrospective cohort study comparing patient characteristics among MRSA-colonized and MRSA-infected infants, rates of MRSA infection before and after screening with targeted decolonization, and MRSA infection among those receiving single or combined decolonization agents.

**Setting:**

Texas Children’s Hospital Pavilion for Women is a 42-bed level three neonatal intensive care units (NICU) in Houston, TX.

**Patients:**

Neonates admitted to the NICU from 2012 to 2022 were included in analysis of MRSA colonization and infection. The gestational age ranged from 22 weeks to 42 weeks.

**Interventions:**

The MRSA screening methodology consisted of weekly surveillance PCR or culture on admission until discharge. If positive, infants underwent decolonization consisting of topical intranasal mupirocin, and if meeting the gestational and chronological age-based criteria, topical 2% chlorhexidine wipes and topical intranasal mupirocin.

**Results:**

The MRSA colonization rate from 2016 to 2022 was 2.2%. Following the screening and decolonization protocol initiated in 2016, there was a sustained downtrend in the rate of MRSA infection. No MRSA-colonized neonates who received both topical mupirocin and Chlorhexidine gluconate (CHG) developed MRSA infection.

**Conclusions:**

We observed a decreased rate of MRSA infection in the NICU following implementation of an MRSA screening and decolonization protocol. While our data suggests that the combination of mupirocin and CHG might prevent infection, further studies are needed due to the low prevalence of MRSA infection in our cohort.

## Introduction

Neonates are at high risk of devastating outcomes from *Staphylococcus aureus* (SA) infection. Methicillin-resistant *Staphylococcus aureus* (MRSA) infections in neonates increases length of stay, morbidity, mortality, and healthcare cost.^1,2^ Infection prevention practices in neonatal intensive care units (NICUs) must address unique challenges associated with this vulnerable patient population, such as extreme prematurity, low birthweight, prolonged hospital courses, use of numerous medical devices, procedures, shared areas or bed spaces, and family visitation practices. One study, published by the Chicago-Area Neonatal MRSA Working Group, describes the epidemiology of MRSA in NICUs with a consensus statement that provides guidance on preventing MRSA infections in NICUs.^3^ The experiences of MRSA colonization and infection in various NICUs in the last two decades have contributed to our understanding of risk factors for MRSA colonization, infection, and outcomes in infants in NICUs.^4–7^

The first published case of MRSA infection in a NICU^8^ dates back to the early 1980s. Since the emergence of community-acquired MRSA in the late 1990s, both healthcare associated (HA) and community acquired (CA) strains can be transmitted to hospitalized neonates.^9,10^ Reich et al found a higher risk of infection associated with HA-MRSA colonization.^4^ In a large meta-analysis, Zervou et al. found that the relative risk of MRSA-colonized patients developing an MRSA infection during the hospitalization was 24.2 among patients admitted to NICUs and pediatric intensive care units, with the MRSA colonization rate among NICU patients being as high as 6.1%.^11^ Healthcare providers and families are both sources of possible transmission of MRSA to the neonate.^12–14^Additionally, vertical transmission from the vaginal canal during delivery is reported to occur.^15^ Risk factors associated with MRSA colonization among NICU patients include very low birth weight, extreme prematurity, prolonged mechanical ventilation, repeated peripheral intravenous line placements, visitors sharing rooming/toileting and a high point prevalence of colonization in the unit.^5,6,10^ Some authors reported that infants with MRSA infection became colonized earlier in the hospitalization, had a longer length of stay, and were less likely to have received mupirocin compared to the neonates who did not develop an infection.^7,11^

Infection control practices form the foundation for preventing transmission of MRSA within NICUs. These include hand hygiene, isolation of positive patients, cohorting, and the use of contact precautions (gown and gloves) when handling positive patients.^3^ Some centers also employ surveillance and decolonization. Topical antimicrobials such as chlorhexidine (disrupts bacterial cell membrane)^16^ and mupirocin (inhibits bacterial protein and RNA synthesis)^17^ are utilized against SA. Decolonization practices vary by institution. A multi-center retrospective cohort study of tertiary care NICUs found that the adjusted hazard of gram-positive cocci infection was 64% lower among neonates who received mupirocin compared to neonates who did not, without increasing the risk of subsequent infections with gram-negative organisms.^18^ Nelson et al. conducted a randomized placebo-controlled study comparing mupirocin and placebo, revealing that up to 80% of neonates treated with mupirocin were successfully decolonized within two weeks with a significant reduction in the rate of invasive SA infections.^19^

Despite improved outcomes with decolonization, some infants develop infection despite negative surveillance cultures. This is likely multifactorial in that MRSA may colonize body sites not sampled, infections can occur without prior colonization and that colonization can occur in between surveillance testing dates.^20^ Goldstein et al. studied the frequency of surveillance by culture methods and found that increasing monitoring from every four weeks to weekly decreased the mean number of MRSA-colonized neonates from 2.9 to 0.6, respectively, thus supporting the use of weekly monitoring.^21^

Recolonization with MRSA after decolonization is more likely to occur in neonates with prolonged hospitalizations and those colonized with mupirocin-resistant strains.^22^ Resistance to mupirocin is a growing concern and is an area under study, particularly in cases of MRSA outbreaks in NICUs that employ decolonization with mupirocin. Balamohan et al. found that decolonization by mupirocin during their study period reduced rates of Methicillin-susceptible Staphylococcus aureus (MSSA) infection but did not impact MRSA infection rates, and that the MRSA strain circulating through the NICU was mupirocin resistant.^6^ In contrast, Arora et al. did not observe higher MICs to mupirocin over a year-long study period of universal decolonization,^23^ suggesting that the widespread use of the topical antimicrobial may not necessarily lead to the emergence of more resistant strains.

The Centers for Disease Control and Prevention Recommendations for Prevention and Control of Infections in Neonatal Intensive Care Unit Patients (2020) include guidelines on screening and decolonization practices.^24^ Consistent with these available guidelines, most NICUs screen for MRSA colonization during an outbreak, however there is variability in decolonization practices. The guidelines currently have a conditional recommendation to consider targeted decolonization for SA-colonized neonates, but no recommendation for the optimal decolonization agent or combination of agents. Thus, we sought to evaluate this in our study.

## Methods

The study setting is a 42-bed level three neonatal intensive care unit in Houston, Texas that has approximately 1000 admissions per year. The Pavilion for Women (PFW) NICU opened in April 2012. This is a single-center retrospective observational study of neonates admitted to the NICU from 2012-2022. Neonates are admitted to private patient rooms with space for rooming in of parents and families. Most neonates are admitted from labor and delivery of the PFW; however, some are transferred from community hospitals due to extreme prematurity.

The surveillance and decolonization protocol initiated in late October of 2016, consists of screening on birth or admission and weekly thereafter until discharge or until MRSA-positive detection by PCR (Xpert MRSA NxG, Cepheid, Sunnyvale, CA). Surveillance specimens are collected by swabbing the nares, axillae, and groin. Prior to 2017, MRSA was detected by culture method only. Starting in 2017, PCR (Xpert MRSA NxG, Cepheid, Sunnyvale, CA) was introduced for MRSA surveillance. If positive, reflex to culture was done to perform susceptibility and further characterization of isolates. MRSA-colonized neonates are placed in contact precautions and undergo decolonization. All MRSA-colonized neonates, regardless of gestational or postnatal age, receive topical mupirocin to anterior nares twice daily for five days. Only neonates who are at least four weeks postnatal age or 36 weeks gestational age (whichever comes first) meet the criteria to receive 2% chlorhexidine wipes, due to limited data supporting safety in premature neonates with compromised skin integrity. The protocol does not include re-screening MRSA-colonized infants after they are decolonized.

MRSA colonization is defined as the detection or isolation of MRSA by either PCR or culture from nasal, axilla, or groin swabs. MRSA infection is identified as the isolation of MRSA by culture from blood, wound, urine, respiratory samples, tissue, or other sites in the presence of clinical illness.

To compare the rates of MRSA infection before and after the implementation of the MRSA surveillance and decolonization protocol in October 2016, the pre- and post-intervention rate of MRSA infection per 1000 patient days was calculated, allowing for a grace period from October 2016 through February 2017 (to account for the time required to train staff, implement order sets in the electronic medical record, etc.). Multiple positive specimens from the same site were considered the same infection if occurring within a 14-day window. Infection rate was calculated as the number of unique infections per 1000 patient days. We calculated the rates of MRSA infection from April 2012 (opening of PFW NICU) through March 2022. Trends of MRSA infection pre- and post-intervention (decolonization protocol) were evaluated using the interrupted time-series analysis (ITSA) regression for single group^25,26^ and depicted by the line and scatter plots. MRSA infection rates at individual time points were also analyzed. All other analyses were performed on Stata version 18.5 (StataCorp LLC, College Station, TX, USA). A p value of <0.05 was considered statistically significant.

Demographic data and individual patient characteristics were compared between neonates who were only MRSA-colonized and those that were MRSA-colonized then developed infections during the time period from October 2016 to March 2022. We then compared MRSA infection between neonates who underwent full, partial, or no decolonization from 2016-2022. Full decolonization was defined as receiving both mupirocin and Chlorhexidine gluconate (CHG) after detection of colonization. Partial decolonization was defined as only receiving mupirocin. The outcome measure, MRSA infection, was compared, and risk ratio (relative risk) was calculated to determine the efficacy of exposures (CHG and mupirocin). This study was approved through the Baylor College of Medicine Institutional Review Board.

## Results

We group our results into population-level epidemiologic MRSA infection data (April 2012–March 2022) and MRSA-colonized patient data (October 2016–March 2022). In total, there were 65 MRSA infections from April 2012 to March 2022. Twenty-six cases of bacteremia (40% of infections) and 23 deep wound infections (35.4%) represented the majority of the infections. Three cases of bacteremia had a secondary site of infection, which were counted as separate infections. Multiple positive specimens from the same site were considered the same infection if both occurred within a 14-day window. Additionally, there were 11 MRSA isolates from body fluid (such as joint spaces/orbital cellulitis/other unspecified sterile sites), 4 cases of pneumonia/empyema (MRSA isolated from respiratory culture in the setting of clinical respiratory change to exclude respiratory colonization), and 1 case of MRSA CNS infection.

**Figure 1.**
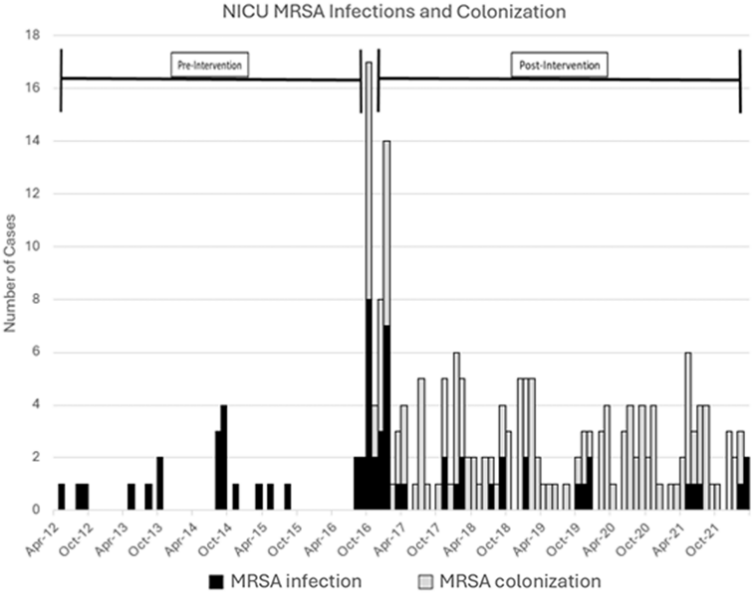
MRSA infection rate (per 1000 patient days) before and after intervention.

**Figure 2.**
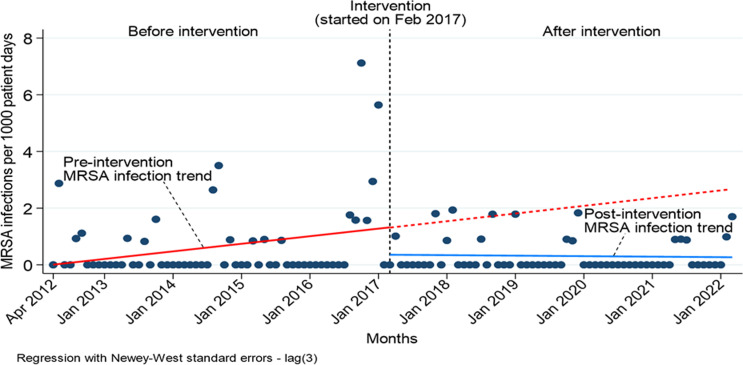
Interrupted time series model comparing MRSA infection rates before and after implementation of surveillance and decolonization protocol.

The rate of MRSA infection per 1000 patient days decreased in the NICU after implementation of the MRSA surveillance and decolonization protocol in October 2016, accounting for a four-month grace period (to allow for NICU staff training on the protocol, building order-sets in the electronic health record and increasing awareness among NICU providers) (Figure 1). Prior to the intervention (April 2012–October 2016), the rate of MRSA infection increased by 0.02 infections per 1000 patient days every month (95% CI −0.01, 0.06, *P* = 0.20). During the first month after full implementation of the intervention, the infection rate decreased by 0.02 infections per 1000 patient days (95% CI −0.06, 0.01, *P* = 0.18) (Table 1). The ITS model shows a sustained monthly rate decrease of 0.97 infections per 1000 patient days (95% CI −2.38, 0.45, *P* = 0.18) (Figure 2).

**Table 1.** Monthly rate for MRSA, pre- and post- intervention

	Monthly rate, per 1000 patient days (95% CI)^*^	p-value for trend^*^
Pre-intervention	0.02 (−0.01, 0.06)	0.20
Post-intervention	−0.97 (−2.38, 0.45)	0.18
Decrease in MRSA rate, 1st month post-intervention	−0.02 (−0.06, 0.01)	0.18
Intercept	0.006 (−0.72, 0.73)	--

* Obtained from the ITSA model^25,26^

There were 5,452 neonates admitted to the NICU from the date of intervention (October 2016) through March 2022. During this period, 120 were MRSA-colonized. The MRSA colonization rate from October 2016 through March 2022 was 2.2%.

From October 2016-March 2022, 104 neonates were MRSA-colonized only (no infection) and 16 neonates were MRSA-colonized and subsequently developed MRSA infection. The patient characteristics of neonates who were only MRSA-colonized and those who were MRSA-colonized and developed infection are outlined in Table 2. The median birth weight of MRSA-colonized neonates who developed infection was 897.5 grams (interquartile range IQR 697.5, 1145) compared to the MRSA-colonized neonates who did not develop infection (1380 grams (IQR 1007.5, 1995), *P* < 0.001. The MRSA-colonized neonates who developed MRSA infection were of younger gestational age than those MRSA-colonized neonates who did not develop infection, with median age 27 weeks compared to 30.1 weeks, respectively (*P* < 0.001). The time to colonization from birth did not show a statistically significant difference between the two groups of neonates. Colonized neonates who developed infection also had a longer length of stay (median 65.5 d) compared to neonates who were only MRSA-colonized (median 42 d), *P* <0.001. Four out of the sixteen MRSA-infected neonates were born at another institution, whereas two out of the one hundred four MRSA-colonized neonates were out-born. There were no statistically significant differences between the two groups of neonates in terms of delivery method, gestation quantity, or sex. Three of the sixteen MRSA-colonized neonates who developed infection died (including one who had both MRSA and *Klebsiella* bacteremia).

**Table 2.** Patient characteristics

Characteristic	MRSA-Colonized only	Colonized then Infected	
	(n = 104)	(n = 16)	p-value
Birthweight (grams), median (Q1, Q3)	1380.0 (1007.5, 1995.0)	897.5 (697.5, 1145.0)	<0.001
Gestational age (days), median (Q1, Q3)	30.1 (28.1, 34.2)	27.0 (25.4, 28.9)	<0.001
Time to infection (days), median (Q1, Q3)	N/A	22.5 (11.0, 51.5)	N/A
Time to colonization (days), median (Q1, Q3)	13.0 (7.0, 21.0)	7.0 (5.0, 19.0)	0.38
Length of stay (days), median (Q1, Q3)	42.0 (15.0, 57.0)	65.5 (56.0, 113.5)	<0.001
Male sex, n (%)	56 (53.8)	7 (43.8)	0.45
Inborn, n (%)	102 (98.1)	12 (75.0)	<0.001
Multiple gestation (vs single live newborn), n (%)	37 (35.6)	3 (18.8)	0.26
C-section delivery (vs vaginal), n (%)	68 (65.4)	0 (0.0)	<0.001
Mortality (any cause), n (%)	2 (1.9)	3 (18.8)	0.017

In comparing the outcome of MRSA infection in neonates who received full, partial or no decolonization treatment (Table 3), among the 120 MRSA-colonized neonates, 19 received both mupirocin and CHG, 89 received only mupirocin, and 12 neonates received neither agent due to being discharged or transferred out of the unit prior to decolonization. None of the neonates who received both mupirocin and CHG developed MRSA infection. Fourteen of 89 (15.7%) neonates who received only mupirocin developed MRSA infection. Two of 12 (16.7 %) of neonates who did not receive any decolonizing agent developed MRSA infection. In comparing MRSA infection outcomes between neonates who received only mupirocin (due to age) vs neonates who did not receive mupirocin, the risk ratio is 0.78, 95% (CI 0.20, 3.02) (Table 4). In comparing the neonates who received at least one decolonizing agent (mupirocin alone or combination of mupirocin and CHG), vs those who received neither, the risk ratio of developing MRSA infection is 0.94, 95% (CI 0.24, 3.65) (Table 5).

**Table 3.** Comparing MRSA infection among neonates who received different combinations of decolonizing agents

	Not decolonized	Mupirocin only	CHG + mupirocin	Total n
**MRSA Infected**	2	14	0	16
**Not MRSA Infected**	10	75	19	104
**Total**	12	89	19	120
**Infection rate**	2/12 = 16.7%	14/89 = 15.7%	0/19 = 0%	

**Table 4.** Comparing MRSA infection outcome among neonates who received any decolonization agent or none

	MRSA Infection	No MRSA Infection
Mupirocin/Mupirocin + CHG	14	94
None	2	10

MRSA infection among neonates who received either mupirocin alone or mupirocin + CHG (RR 0.78, 95% CI 0.20, 3.02)

**Table 5.** Comparing MRSA infection among neonates who received any mupirocin vs no mupirocin

	MRSA Infection	No MRSA Infection
Only mupirocin	14	75
No mupirocin	2	10

MRSA infection among neonates who received only mupirocin (due to age) compared to those did not receive mupirocin (RR 0.94, 95% CI 0.24, 3.65)

## Discussion

Four decades since the first published case of MRSA infection in a NICU,^8^ MRSA has become endemic across centers that hospitalize very premature neonates. In response to an increased incidence of MRSA colonization and infection in this NICU, we implemented decolonization in addition to evidence-based infection control and prevention measures. The epidemiologic curve (Figure 1) and ITSA demonstrate that while we continue to identify MRSA colonized infants in the unit, rates of MRSA infection have declined after implementation of the MRSA surveillance and decolonization protocol. Furthermore, we have not had clusters or outbreaks of MRSA infections since implementing the protocol.

Following implementation of surveillance and decolonization protocol the MRSA colonization rate of 2.2% is comparable to that reported in other NICUs that care for extremely premature infants. Consistent with National Guidelines^24^, our NICU employs targeted decolonization for all MRSA-colonized neonates. To our knowledge, this is the first study that attempted to compare the efficacy of decolonization by mupirocin alone vs mupirocin and CHG wipes at preventing MRSA infection and found that none of the neonates who received the combination of mupirocin and CHG developed MRSA infection. However, we were unable to reach a statistically significant conclusion in comparing the decolonization methods, possibly due to the low prevalence of MRSA infection in the cohort.

As reported in the literature, we found that lower birth weight, lower gestational age and longer length of stay were significant risk factors for developing MRSA infection. This would support the role that regular surveillance has on mitigating spread of MRSA and decreasing the colonization burden among patients at any given time. The median time to colonization is 13 days among MRSA-colonized only and 7 days among colonized neonates who developed infection (*P*= 0.38), which may be important as the frequency of surveillance varies by institution. We advocate for weekly screening to identify colonized neonates thus intervening with infection prevention methods such as contact isolation precautions, cohorting, and decolonization to prevent horizontal transmission.

This is a single center study from Texas Children’s Hospital, therefore results may not be generalizable to other centers. Due to the retrospective nature, we could not account for variability among specimen collection. We included only those MRSA infections diagnosed by culture methods, potentially missing infections identified by PCR methodology from sterile sites. Due to the protocol design, infants who are MRSA-colonized then underwent the decolonization regimen were not re-screened for MRSA therefore our study could not determine if decolonization is enduring. Prematurity could serve as a confounder in assessing efficacy of decolonization as it both increases SA infection risk and may serve as an exclusion for receiving CHG. The ITSA does not account for natural changes in the epidemiology of SA infections, including a potential decrease in MRSA infections in the post intervention period. We do not have existing data on staff adherence to personal protective equipment, isolation compliance or the role of parental colonization.

Our study showed a decrease in neonatal MRSA infections following implementation of a MRSA screening and decolonization protocol. Future studies should evaluate the durability of decolonization as well as the development of mupirocin and CHG resistance. Lastly, impact of a similar protocol on MSSA infection rates in the NICU warrants evaluation.
